# A Safety Climate Framework for Improving Health and Safety in the Indonesian Construction Industry

**DOI:** 10.3390/ijerph17207462

**Published:** 2020-10-14

**Authors:** Fatma Lestari, Riza Yosia Sunindijo, Martin Loosemore, Yuni Kusminanti, Baiduri Widanarko

**Affiliations:** 1 Occupational Health & Safety Department, Faculty of Public Health, Universitas Indonesia, Depok 16424, Indonesia; baiduri@ui.ac.id; 2 Faculty of Built Environment, UNSW Sydney, Sydney, NSW 2052, Australia; r.sunindijo@unsw.edu.au; 3 School of Built Environment, University of Technology Sydney, Sydney, NSW 2007, Australia; martin.loosemore@uts.edu.au; 4 Occupational Safety, Health & Environmental Unit, Universitas Indonesia, Depok 16424, Indonesia; yekananti@gmail.com

**Keywords:** construction, Indonesia, locus of control, safety climate, safety norms

## Abstract

The Indonesian construction industry is the second largest in Asia and accounts for over 30% of all occupational injuries in the country. Despite the size of the industry, there is a lack of safety research in this context. This research, therefore, aims to assess safety climate and develop a framework to improve safety in the Indonesian construction industry. Quantitative and qualitative data were collected from 311 construction workers. The results show a moderately healthy safety climate but reflect numerous problems, particularly around perceived conflicts between production and safety logics, cost trade-offs being made against other competing project priorities, poor safety communication, poor working conditions, acceptance of poor safety as the norm, poor reporting and monitoring practices, poor training and a risky and unsupportive working environment which prevents workers from operating safely. Two new safety climate paradoxes are also revealed: contradictions between management communications and management practices; contradictions between worker concern for safety and their low sense of personal accountability and empowerment for acting to reduce these risks. A low locus of control over safety is also identified as a significant problem which is related to prevailing Indonesian cultural norms and poor safety policy implementation and potential conflicts between formal and informal safety norms, practices and procedures. Drawing on these findings, a new integrated framework of safety climate is presented to improve safety performance in the Indonesian construction industry.

## 1. Introduction

The Indonesian construction industry has been growing rapidly in recent years due to the government’s focus on infrastructure development. It is a significant industry which contributed about 10.3% to the country’s gross domestic product (GDP) in 2018, making it the second largest construction market in Asia [1]. The industry employed 4.8 million people in 2010 and the number increased drastically to 8.1 million in 2017, representing about 6.7% of the workforce in Indonesia. This rapid growth is predicted to continue [2]. Despite its significance, the Indonesian construction industry has a poor occupational health and safety (OHS) performance record. Statistics on the number and rate of accidents in the construction industry are unreliable, but it is estimated that 30% of all occupational accidents in Indonesia occur in the industry [3]. However, research on OHS in the Indonesian construction industry remains scant and can be divided into two main areas: research which identifies safety risks, influences and consequences; and research which explores the industry’s safety culture and climate. 

This paper contributes to the second theme, building on the limited number of previous studies in this area. For example, Sutalaksana and Syaifullah [4] found that positive safety climate in the Indonesia construction sector positively influences safe behavior because it reduces work pressure and removes barriers to work safely. Andi [5] determined the level of safety culture in the Indonesian construction industry using six factors, including top management commitment, safety communication, safety rules and procedures, work environment, worker competence, and worker involvement. In high-rise buildings in Indonesia, Irawadi [6] suggested that safety climate is a predictor of safe behavior and positively influences project performance. Most recently, Machfudiyanto and Latief [7] have also recently developed a conceptual framework for developing construction safety culture in Indonesia. 

While valuable, the problem with this research is that it is fragmented and has sometimes failed to distinguish between the related but distinct concepts of safety culture and safety climate (see for example Andi [5]). Furthermore, previous research on safety climate in Indonesia has not considered the multilevel nature of safety climate [8]. Finally, while valuable in highlighting the importance of safety climate as a key variable in safety performance, there is as yet no integrated framework to guide future research and management action in this area. To address this gap in knowledge, this paper aims to develop a safety climate framework for the Indonesian construction industry. 

More specifically it aims to do this by exploring three key research questions which have so far gone unanswered:

1. What is the state of safety climate in the Indonesian construction industry?
2. What are the strengths and weaknesses of this climate?
3. What can be done to improve it?

Such research is important because as Lingard et al. [9], Sunindijo and Zou [10] and Hecker and Goldenhar [11] indicate, the development of a safety climate framework could potentially act as an important leading indicator for better safety outcomes in the industry. Furthermore, Andersen et al. [12] showed that a positive safety climate at both the construction site level and workgroup level was associated with lower rates of self-reported accident, improve safety performance and decreased risk of accidents and injuries.

## 2. Safety Climate 

Safety climate was first defined by Zohar ([13] p. 96) as “a summary of molar perceptions that employees share about their work environments.” Criticizing the prevailing tendency for managers to blame workers for poor safety, Zohar’s contribution was to reposition arguments around the need for management to improve safety climate, measured using a safety climate survey which explored factors other than the typical safety risks which had dominated safety research until that time. Building on this formative work, many definitions of safety climate have been proposed. For example, Cooper and Phillips ([14] p. 497) defined safety climate as the “shared employee perceptions of how safety management is being operationalized in the workplace at a particular moment in time”, while more recently, WorkCover Queensland [15] defined safety climate as “the perceived value placed on safety in an organization at a particular point in time”. 

The term safety climate is often used interchangeably with the term safety culture [16]. Helpful in distinguishing between safety culture and climate is the definition advanced by Health and Safety Executive [17] which contained three interrelated dimensions of safety culture: behavioral; corporate; and psychological. The behavioral dimension is concerned with what people do within the organization, which includes the safety-related activities, actions, and behaviors exhibited by employees. The corporate dimension is reflected in the organization’s policies, work procedures, management systems, control systems, and communication flows. The psychological dimension of safety culture concerns how people feel about safety and safety management systems and is what is commonly referred to as the safety climate of the organization, showing that safety climate is an integral part of safety culture [18]. As Moran and Volkweln [19] note, organizational climate refers to the recurring patterns of behavior, attitudes and feelings that are visibly perceived to characterize life in an organization by its members. When individual perceptions of the impact of a work environment on their well-being (psychological climate) are shared among members of an organization, these collective perceptions represent the organizational safety climate. In contrast, organization culture refers to the deep, stable and invisible patterns of collective values, beliefs, assumptions, norms and expectations on which people’s behavior, attitudes and feelings depends [20]. Research also shows that safety climate is a multilevel construct with distinct sub-climates operating both vertically and horizontally within different work groups and teams and levels of organization [9,21]. 

Contemporary safety research, within and outside the construction industry, considers safety climate as a valid concept and reliable indicator of OHS performance [22,23,24]. There are numerous benefits of using safety climate as an indicator of safety performance [16,25,26]. First, traditional safety indicators, such as incidence rates, are lagging indicators, measuring the performance of the past based on what has already happened. This limits managers’ ability to proactively manage risks before they arise and their accuracy also depends on the capability and honesty of an organization in reporting incidents. Furthermore, organizations that diligently report and investigate incidents are disadvantaged in comparison to those that are careless in their reporting, making it hard to motivate organizations to accurately report their incident cases [27]. Safety climate, on the other hand, is a leading indicator of safety performance which is able to identify areas of risk in advance, allowing organizations to proactively improve them. Importantly, the measurement of safety climate in an inherently collaborative process requiring the involvement of employees, which not only utilizes an important source of human and intellectual capital too often neglected in traditional lagging indicators, but in itself, contributes to enhancing safety climate [16,25,26].

In seeking to develop a tool to measure safety climate, there has been a proliferation of research seeking to identify safety climate dimensions. In an early attempt to synthesize and simplify this literature, Guldenmund [18] suggested that the number of safety climate dimensions can be reduced significantly by relabeling existing dimensions. Zohar and Luria [21] distinguished between strong and weak safety climate, with strong safety climate being characterized by a high degree of consensus between members about commitment to safety, while weak safety climate are characterized by a low level of consensus. Hecker and Goldenhar [11] concluded that the major interrelated variables used to identify safety climate in the research literature include; management commitment; employee involvement and/or empowerment; safety communication; safety competence; balance of safety and production; and supervisory and co-worker safety support. They also noted that construction is a highly segmented industry and that efforts to measure and improve safety climate and safety culture may require different strategies for different types of companies, large and small. In later work, Schwatka et al. [28] review of construction-specific articles that developed and/or measured safety climate, confirmed that researchers commonly define safety climate as perception-based, but that the indicators used to measure safety climate varied widely, with safety policies, procedures, and practices being the most common, followed by general management commitment to safety. Furthermore, they found that safety climate scores were commonly compared between groups (e.g., management and workers, different trades), and often correlated with subjective measures of safety behavior rather than objective measures of safety and health outcomes. The most recent construction research to develop a specific tool to measure safety climate is Zou and Sunindijo [16] who found that safety climate is generally measured in relation to six key factors: degree of management commitment to health and safety; effectiveness of informal and formal communication between managers and the workforce about health and safety issues; safety policy, rules, and procedures that are perceived as practical, realistic, and appropriate; a supportive environment characterized by safe working conditions and trusting relationships between workers and managers; a workforce that is actively involved in developing health and safety initiatives rather than being passive recipients of safety policy and procedures from the top; and regular training for employees to identify safety risks and perform their works safely. 

## 3. Method

### 3.1. Survey Instrument Development

This research adopted a mixed method research methodology. Method triangulation, which refers to the use of multiple data collection methods to observe the same phenomenon [29], was used in this research to gain a deeper understanding when analyzing the data. Informed by Zou and Sunindijo’s [16] safety climate framework, a face-to-face survey instrument was developed. The survey has three sections. The first section collected demographic data, including age, gender, level of education, and years of working in the construction industry. The second section consisted of 58 questions to assess six dimensions of safety climate based on the Zou and Sunindijo’s [16] framework. A third section provided respondents with the opportunity to volunteer qualitative feedback about their perceptions of safety climate on their projects. 

In constructing the survey, we were acutely aware that Zou and Sunindijo’s [16] safety climate framework had been developed and tested in Australia rather than Indonesia (although it was based on previous climate research in various contexts). This is important since national culture influences attitudes towards OHS [30] and survey questions can often lose meaning in translation from English to Indonesian. Therefore, before being administered, it was pilot tested on a small sample of respondents to ensure that the items were not ambiguous. As a result of this pilot study the wording of some questions were revised.

### 3.2. Data Collection

The face-to-face survey was undertaken with construction workers, supervisors and managers randomly sampled from three purposefully sampled construction sites managed by tier-one contractors in Indonesia. While we acknowledge that there is a need to conduct research in other tiers of the Indonesian construction industry, the paucity of research in this area means that empirical evidence and focus is a priority in this early exploratory phase of construction safety climate research. To this end, construction sites managed by tier-one contractors are typically larger and offer more respondents in one location. Furthermore, research in this context provides a useful benchmark against which future research in other strata of the Indonesian construction industry can be compared. 

In choosing construction projects as our unit of analysis, we also acknowledge research which shows that there can be more than one type of safety climate in an organization, e.g., one type at the organizational level and another at the project level [9,21]. Further research is therefore also needed at organizational level. 

We used face-to-face survey administration to maximize our response rate and because this is the most reliable way to contact construction workers in Indonesia. An electronic survey was not feasible since we could not guarantee that potential respondents had access to the computers and the internet. There are also numerous other potential problems with on-line surveys other than access, not least ensuring that they are completed by the targeted respondents [31]. Given the non-English speaking background and varying dialects of many construction workers, it was also important to be able to answer any queries face-to-face in answering our questions. 

To minimize the possibility of social desirability bias, which is a risk in any OHS type research [32,33], the surveys were anonymous and neutrally administered by the researchers with no influence from the respondents’ supervisors. A forced response six-point Likert-scale format, ranging from strongly disagree to strongly agree, was also used. Finally, in accordance with ethics clearance requirements, respondents were provided with information about the aims of the research and why they had been selected. They also could stop their participation and withdraw their data at any time.

All respondents approached by the researchers agreed to participate in the research resulting in the collection of 311 valid responses. Among these, 117 also responded to the open-ended question and gave qualitative feedback. The profile of the respondents is presented in Table 1.

### 3.3. Data Analysis Approach

The qualitative data were analyzed using thematic analysis which involved the researchers ‘structuring’ the narratives by inductively pinpointing, examining, and recording common social structure themes within the data across each survey response. In this case, the data were structured based on the Zou and Sunindijo’s [16] framework. Following Braun and Clarke [34] and Guest [35] the inductive thematic analysis involved several stages starting with: ‘immersion’ in the data (repeatedly reading the interview transcripts to obtain a high level of familiarity with the data); categorization/coding (organizing and generating an initial list of items/codes from the data-set, that have a reoccurring pattern as it relates to the theoretical constructs in our literature review); searching for themes (examining how codes combine to form over-reaching themes which are phrases or sentences that identifies what the data means in relation to the research questions); refining themes (continuing to search for data that supported or refuted proposed themes and connections between overlapping themes). The quantitative data were analyzed descriptively by computing the mean responses of respondents to the 58 safety climate items. The purpose of this analysis is to identify strengths and weaknesses among the safety climate items and dimensions based on the perceptions of the respondents. The descriptive quantitative analysis was used to complement the results of the qualitative data analysis. 

## 4. Results and Discussion

### 4.1. Overall Analysis

Table 2 presents the overall safety climate score and the score of each dimension. The overall mean score of 4.45 (slightly agree to agree) is not a strong result but is surprisingly good given the ‘very poor’ safety record of the Indonesian construction industry. As a comparison, the overall safety climate score in the Australian construction industry, which has much better safety records and strong regulation governance and enforcement, is 4.50 [36], only marginally higher than the overall score in this research. This can be explained in a number of ways. For example, given the strong relationship between safety climate and safety performance found by numerous authors, such as Lingard et al. [9] and Hecker and Goldenhar [11], the results could suggest that the problems of safety performance might be elsewhere in the Indonesian industry (for example—on second and third-tier contractor sites). To explore this explanation, further research is needed on Indonesian sites managed by lower tier contractors.

The means of safety climate dimensions range from 4.20 to 4.82, showing that there are no areas of particular strength or weakness in the Indonesian construction industry’s safety climate. However, these results do indicate that although managers are perceived to be reasonably committed to OHS and to communicate this (the highest ranked dimensions), the formal rules and procedures and informal work environment they put in place (the lowest ranked dimensions), are not perceived to reflect and support that commitment. In other words, there appears to be a conflict between management words and management actions. In exploring this further and in answering the call for new theoretical insights into construction research, which Dainty and Loosemore [37] argue remains largely disconnected from theoretical developments in social and behavioral sciences, this finding highlights the potential value of new institutional theory as an innovative theoretical lens to understand safety in the Indonesian construction industry (and indeed in other contexts where similar results may emerge). New institutional theorists define institutions as forms of social organization (safety rules, practices, normal and procedures), negotiated by organizational stakeholders which become accepted and imbedded over time to produce resilient, stable and recurring patterns of safety behavior [38,39]. These institutions can be both ‘formal’ (codes of conduct, contracts, procedures, policies, laws) and ‘informal’ (norms, practices and narratives) and are reinforced and continuously changing as people go about their work [40]. Formal institutions are defined by their conscious design, formal documentation and by their dissemination and enforcement through official channels, sanctions and rewards. In contrast, informal institutions are invisible, tacit, undocumented and communicated and enforced through an organization’s informal communication networks, power structures and culture and through subtle, hidden, and even illegal channels. However, in contrast to formal institutions which can be easily studied by researchers, because they involve obvious sanctions and actors, informal rules are hard to identify because people may not even realize they exist. This will require future safety climate researchers to engage with more qualitative methods of research such as ethnography, which are specifically designed to untangle such complexities.

### 4.2. Individual Climate Dimension Analysis

In exploring the results in more detail, the following sections discuss each dimension of safety climate in rank order.

#### 4.2.1. Management Commitment

This dimension was the top ranked area overall. Table 3 shows that the respondents perceived that their project managers consider safety as a top priority and act quickly to correct safety problems. However, there are contradictions in the results, since managers were also perceived to be inconsistent in their attention to safety, and to not express concern if safety procedures were not followed. These results suggest that unsafe conditions are tolerated on site and that workers do not feel that managers care about their safety, even if they profess to do so.

In their qualitative feedback, respondents acknowledged the importance and strength of management commitment and also the inconsistencies in our quantitative results.

Even though it’s not optimum yet, the implementation of health and safety programs in the workplace is quite good. The motto ‘safety first’ should be maintained.
Management should prioritize OHS standards and procedures at work to minimize occupational accidents.
Management should pay more attention to health and safety. Management should improve health and safety further.
OHS implementation should be improved further to the right direction.

In improving management commitment, respondents identified three main priorities. First, they wanted the management team to prioritize OHS over production in the projects.

Management must support health and safety programs.
Production and progress are not reasons to neglect health and safety implementation.
Health and safety should not be neglected no matter how important the work is.

Second, sufficient budget is needed to implement OHS and is seen as an important indicator of management commitment.

Health and safety performance depends on health and safety budget and top management commitment.
Health and safety budget needs more attention. Generally, in practice this budget is non-existent, or the amount is small.

Third, management teams should explicitly demonstrate their OHS commitment by being proactive and providing necessary support and resources to implement OHS.

Management should practically implement health and safety, not only theorizing.
Health and safety implementation must be demonstrated in practice, not merely by theory and demanding from those at the lowest level.
Health and safety awareness and commitment must start at the top then to the lower levels.

These results are important since research has found that employees are more willing to cooperate to improve OHS performance when they perceive that their managers care about their personal health and safety [24,41]. This research shows that visible management commitment converts formal OHS rules and policies into organizational values, which is important because corporate values are the fundamental beliefs upon which employee behavior is based [42]. For individual employees, values signpost the importance managers attribute to certain safety behaviors and give employees a sense of what is right or wrong. These values are transmitted through cultural mechanisms such as socialization with managers and other co-workers [43].

The concerns raised by the respondents about conflicts between productivity and safety are especially concerning but supports other recent research which has highlighted this problem of competing institutional production and safety logics (see Smith [44]). According to Crumbley [45], management commitment to an organization’s OHS program communicates the message that safety is valued as a primary priority, even at the expense of productivity. Sunindijo and Zou [46] suggested that management should integrate OHS into project schedule and budget and decision making to demonstrate that OHS is as equally important as traditional project objectives.

The respondent concerns about cost trade-offs being made in relation to safety and other project priorities, have also been raised in other research. For example, in exploring this dilemma in Australia, where law requires adequate investment in OHS, O’Neill ([47] p. 6) argues that “for too long, the business case for investing in measures to ensure the health and safety of workers has been viewed in restrictive, financial terms and based on inadequate and inherently biased data”. In questioning the widespread assumption that safety is bad for productivity, this she argues makes demonstrating the business case for OHS spending problematic. 

In practical terms, according to Crumbley [45] managers can successfully transmit their commitment to safety in the workplace by: providing positive feedback for employees and supervisors using safe work practices; taking time to support safety activities; requiring supervisors to report incident investigations, causal and contributing factors, and the status of corrective actions; participating actively in safety committee meetings and encouraging progress on action items; including safety as an agenda item at regular meetings at all levels; participating in informal inspections; and providing support and participation for safety events and initiatives. These are clearly areas that managers in the Indonesian construction industry need to pay attention to.

#### 4.2.2. Communication

As shown in Table 4, safety communication made the respondents pay attention to safety. However, the results also raise concerns such as availability of managers to discuss safety and the adequacy of methods used to communicate safety information. 

Qualitative feedback pointed to the importance of socialization of OHS and the need to keep up to date with emerging construction technologies which can facilitate safer working.

Socialization of health and safety program should be continued and unit leaders should be appointed to lead health and safety implementation.
The process of purchasing health and safety equipment and tools should be faster. Those on site should be kept updated regarding the availability of health and safety equipment and tools.

These results reflect findings in other countries which shows a direct relationship between safety communications and safety performance [48]. This research shows how complex and multidimensional the challenge of effective OHS communication is. For example, Alsamadni et al. [49] found that the frequency and method of communication are important differentiators between project teams with low and high accident rates. Trajkovski and Loosemore [50] found that language problems across different cultural groups were a serious barrier to safety on Australian construction sites. More recently, Edirisinghe and Lingard [51] and Lingard et al. [52] found that emerging information technologies such as multimedia, video and infographics are often more effective for communicating safety information than conventional methods of written and verbal safety communication. This clearly an area which Indonesian managers could explore in improving their safety climate. 

#### 4.2.3. Training

Table 5 presents the items in the training dimension, which was the third ranked dimension overall. Our results point to particular concerns around being able to identify potentially hazardous situations and a lack of investment in safety training. 

Qualitative feedback emphasized the importance of improving training and the need to make it more practical, relevant and frequent.

Health and safety training is absolutely needed to provide health and safety knowledge to all.
Health and safety briefings should be given more frequently, particularly for those who work in the office and rarely go on site. During the briefings, there should also be practical examples, not only presenting contents.

These findings reflect Nyateka et al. [53] who found that the construction industry invests relatively little in safety training. For example, Safe Work Australia [54] found that 39% of construction employers did not provide any work health and safety training to their employees. This is despite a considerable body of research, which has identified training as a critical factor in influencing the attitudes of construction workers towards safe behavior on construction sites. For example, Lingard and Yesilyurt [55] found that first aid training made participants more aware that their own behavior as an important factor in the avoidance of occupational injury. Teo et al. [56] advocated using technical safety training and behavioral conditioning techniques to improve construction safety and Albert and Hallowell [57] and Oswald et al. [58] found that health and safety training programs improve employee compliance with health and safety requirements. Rodríguez-Garzón et al. [59] and Namian et al. [60] also found that training had a significant impact on perceptions of safety risk. 

While training has been identified as an important dimension of safety performance, questions have also been raised about the nature of this relationship. For example, Trajkovski and Loosemore [50] found that compulsory safety training in the Australian construction industry is often ineffective for construction workers from minority cultural backgrounds and Bahn and Barratt–Pugh [61] found that while safety induction training in Australia is perceived to have a positive effect on safety culture, there is a need for more robust and regulated training that is regularly repeated. Most recently, Malouf and Loosemore [62] argued that while there has been a large amount of research on construction safety training and its impact on positive safety attitudes, much of the evidence has been anecdotal. They found that construction workers and managers emerge from this training with slightly better knowledge of safety risks, a better intention to behave safely but not caring any more about the issue. Anderson et al. [12] support this and conclude that safety climate can be improved by using multifaceted and integrated safety training programs, which target workgroups values, beliefs, and behavior as well as management practice. In Indonesia, this is also reflected by Endroyo et al. [63] who recommended that OHS training in Indonesian construction should have four components: (1) OHS theory and application; (2) adoption of competency-based, cooperative, and contextual learning in teaching and learning; (3) supportive tools, equipment and environment for learning; and (4) portfolio learning evaluation. 

#### 4.2.4. Personal Accountability

Personal accountability was the fourth ranked dimension of safety climate. As presented in Table 6, the respondents agreed that a safe place to work is meaningful and safety is their number one priority when completing a job. Despite recognizing the importance of safety, they felt worried about being injured and could not influence safety at work. The simply followed instructions from their supervisors or managers without referring back to safety policy and procedures. They were also not willing to report co-workers who work unsafely.

Qualitative feedback pointed to a widespread acknowledgement of personal responsibility for one’s own safety.

Health and safety is the responsibility of all, not only the responsibility of safety personnel.
I am responsible to work safely according to procedures.
Health and safety starts from oneself.

These findings are interesting and indicate an inherent paradox within the Indonesian construction workforce—while OHS was centrally important to the respondents, they felt a lack of personal accountability and empowerment to mitigate this risk. It is interesting that some of these findings reflect research in other countries. For example, a major survey by Safe Work Australia [54] found that 25% of all construction workers indicated that they accepted risk-taking at work as the norm and that there is a high chance of injury. Loosemore and Lam [64] found that while the locus of control (self-perceived influence over safety decision-making) is high in relation to health and safety issues, there is considerable discrepancy in perceived levels of influence between different occupational, gender and ethnic groups. Their findings relating to cultural diversity are important and hint at explanations as to why Indonesian workers feel disempowered. Here Hofstedes’ work is especially relevant in showing the relatively high power-distance culture in Indonesia [65] where it is culturally considered unacceptable to question those in the position of authority [66]. 

Hofstede’s work is also useful in explaining why Indonesian workers are unlikely to report the unsafe practices of co-workers, even if it places them at risk personally. In Hofstede’s research, Indonesia is classified as a collectivist culture where people emphasize work group goals above individual needs or desires [65]. Furthermore, recent research by Smith [44] points to the tensions that can exist between the production and safety institutional logics that exist in construction projects which coupled with Indonesia’s collectivist culture, is likely to mean that workers will tend to follow the orders of supervisors to complete the job within budget and program and if necessary put personal safety aside as a secondary consideration. 

These findings link back to our earlier findings in relation to management commitment, communications and training where our results indicate that construction workers in Indonesia are not encouraged to speak-up about safety risks, to report incidents and the unsafe behaviors of co-workers and are not given the knowledge to be able to identify these problems when they arise. As Lobel [67] found, employees will choose to report when they feel empowered and when they perceive that the employer takes their opinions seriously. This highlights the importance of supervisors in encouraging personal accountability for safety [68]. Research has proposed strategies to overcome these reasons for not reporting, including cultivating a climate of no blame, open communication, and building trust and positive supervisor-subordinate relationships [69]. 

#### 4.2.5. Rules and Procedures

Within the rules and procedures dimension, the respondents agreed that safety procedures are followed by all as shown in Table 7. However, the respondents also perceived that safety rules and procedures are difficult to understand and impractical. There is also an indication that safety rules and procedures are ignored to meet project objectives.

The feedback from the respondents indicates that enforcement and sanctions are critical to ensure that OHS rules and procedures are followed.

Facilities and equipment to support health and safety are already quite good. Its enforcement needs to be improved.
Health and safety must be implemented firmly so that work is done according to the policy.
If an organization does not implement health and safety at work or in a project, there should be heavy sanctions as stipulated in OHS legislation.

Overall the results support other research which shows that the enforcement of OHS regulations by governments in developing countries like Indonesia are often lacking [70]. As Umeokafor et al. [71] note, safety regulations and rules without proper enforcement are tantamount to no laws at all. Our findings open up new and important unanswered questions about the interactions between formal and informal rules in an OHS setting, since without the proper enforcement of formal OHS rules, it is likely that informal rules will develop to substitute for them. For example, while this has not been explored in the field of construction safety, in other areas like gender studies, construction researchers have begun to explore how informal rules and practices intersect with, compete with, subvert or even substitute for formal policies [72]. The potential value of this research in exploring our results further, is that it also recognizes that the design of safety policies is the result of multiple designers with different interests and perspectives and that policy designers face constraints and challenges in the creation and implementation of new rules and procedures. This is because organizations have varying absorptive capacity (especially if there is poor safety training and education—see Loosemore and Malouf [62]) and can also deflect them (if for example they compete with other institutional logics like production as they do in construction—see Jia et al. [73]). This research also points to the importance of ‘robustness’ and ‘revisability’ in policy design [74]. Robustness refers to the resilience of policy over time and is linked to clarity of values underpinning them and the nature and effectiveness of policy enforcement (both being problems raised earlier in relation to our findings about management commitment). Revisability is the capacity for policy adjustment in the face of implementation challenges. This can be problematic when safety policies originate in head office without consideration of their practical implementation on site [32]. In this context, it is notable that respondents complained that policies were not practical and were difficult to understand. Our results therefore indicate that there are problems in both OHS policy implementation and design which need to be resolved in the Indonesian construction industry. 

#### 4.2.6. Supportive Environment

The supportiveness of the environment to OHS was the lowest ranked dimension. Many areas of concern are raised in this dimension as shown in Table 8, the most significant being: the work environment creating a high possibility of accidents; working conditions making it difficult to work safely; work targets not aligning with safety targets; I am given enough time to get the job done; and the lack of tools and equipment to do a job safely. 

The most frequently raised issue in the qualitative feedback was the lack of availability and poor condition of personal protective equipment (PPE).

PPE should be made available, including gloves, dust masks and socks.
PPE should be ready before project activities start.
Many work boots are broken.
Lack of attention to PPE, such as broken shoes and broken vest.
The number of safety equipment and tools should be increased, such as full-body harnesses, boots, hardhats, vests, etc.
Not all PPE is available.
Please provide more gloves and sunglasses.
PPE is always inadequate.

One respondent said that while safety harnesses were provided, there were limited anchor points to attach those harnesses. Another respondent commented that he felt unsafe even though he was wearing PPE.

Even though wearing a full-body harness is a must, organizations should pay attention to where the harnesses can be anchored. Likewise for installing safety nets.
I want to be completely sure that I am safe when I follow OHS rules. Sometimes I still feel scared even though I wear PPE.

Housekeeping issues were also raised by our respondents. In Indonesia, workers often live on or around construction sites.

Electrical-related safety needs improvements in my workplace because there are many puddles.
The road is dusty, please repair it because it can cause respiratory problems. Please improve the conditions so work can be done comfortably, safely and quickly.
Improving safety in the workplace, particularly electrical cables, because there are many puddles.
There is a need for a sick bay for employees who are experiencing health problems.
Lighting for night works is needed. First-aid kits should be refilled. Cleanliness on site and storage should be given more attention.
Worker accommodation is inadequate for living.
Water for taking a bath is inadequate. Many mosquitoes.
Please pay attention to the availability of clean water in worker accommodation.

Other issues relate to poor welfare, working conditions and entitlements.

Salaries should be given on time according to progress.
Healthy food and drink should be provided every morning so workers are healthy, strong and motivated to work.
Raise our salaries.
Pay attention to our general welfare.

The conflicts between production and safety logics discussed above in relation to other climate dimensions (management commitment, personal accountability and rules and procedures) are repeated again in this dimension. However, respondents also point to the perceived risk to personal safety presented by their working environment, reflecting research in many other countries about the risky nature of construction sites [75]. The comments relating to lack of equipment to do their jobs safely support previous work by Alruqi and Hallowell [22] who point to the importance of PPEs but which also found that workers fail to use PPE when they are provided with it. Therefore, management efforts to provide better PPE need to be accompanied by strong monitoring and enforcement regimes, as supported by our respondent’s qualitative feedback. 

Poor housekeeping has also been highlighted as a major contributor of unsafe conditions and accidents in construction projects [70,76]. However, the reasons for the apparent lack of attention to PPE and housekeeping are unclear, have not been widely explored in construction and require further investigation. The very limited research into the use of PPE in construction indicates that this may be due to numerous factors such as: competitive tendering causing preliminary costs and safety allowances in bids to be cut to win jobs, poorly designed PPE that make it difficult to work productively, the macho culture of the industry creating a stigma around wearing PPEs, poor levels of education about the benefits of wearing PPEs, poor enforcement and monitoring of PPE use, lack of quality, standardization and availability of equipment and PPEs, impacts on self-efficacy and job satisfaction, workplace cultures which make unsafe working the norm and the risk-taking personalities of people who tend to work in the construction industry [77,78,79]. 

The accommodation issues raised by our respondents and other problems such as access to clean water and withholding wages have also been identified as a problem in other studies in developing countries. See for example recent scandals surrounding modern slavery in developing countries [80,81]. Wahyu [82] reports that construction organizations in Indonesia tend to use informal workers to reduce costs and they are often denied basic employee rights such as insurance, sick pay and sick leave and are treated as disposable assets, which can be easily replaced when they fall sick or are injured. Such workers are not monitored by the government and there is a need to better understand this informal construction sector (sometimes called the grey economy) so that appropriate policies can be developed to improve OHS and wellbeing [83]. 

Finally, lack of attention to construction workers’ wellbeing is a surprisingly under-researched issue [84] compared to the growing body of work on construction professionals’ wellbeing [85,86]. Our findings indicate that this is an important focus for future OHS research in Indonesia.

## 5. Conclusions

Addressing the paucity of research in construction safety the aim of this paper was to study the nature of safety climate in the Indonesian construction industry, its strengths and weaknesses and what can be done to improve it. The objective was to produce a model of safety climate for further empirical testing and for facilitating safety improvements in the Indonesian construction industry. In recognizing the limitations of our findings in that they were produced within the context of tier-one construction sites, our results point to a moderately healthy safety climate. However, without comparative research in other tiers of the Indonesian construction industry, it is difficult to make definitive judgements in absolute terms. More research in clearly needed to replicate our research in other comparative contexts. 

In terms of the strengths and weaknesses of this climate, our research highlights several key issues, particularly around conflicts between production and safety logics, OHS cost trade-offs being made against other competing project priorities, poor safety communication, poor working conditions and acceptance of these as the norm, poor reporting and monitoring practices, poor training and a risky and unsupportive working environment which prevents workers from operating safely. 

Our research has also provided new insights. For example, it has highlighted two new OHS climate paradoxes, the first being the contradictions between management words and management practices and the second being the contradictions between concern for OHS and their low sense of personal accountability and empowerment for acting to reduce these risks. Other new insights relate to the low locus of control of our respondents and how this may be related to prevailing Indonesian cultural norms around high levels of collectivism and power distance. Finally, our results also shine a new light on poor OHS policy enforcement and suggests that this is related to problems of policy complexity, practicality, robustness and revisability and potential conflicts between formal and informal OHS norms, practices and procedures. 

In advancing theory in this area, our research also points to the potential value of new institutional theory in exploring the problematic interactions between formal and informal OHS normal, practices and procedures we have uncovered. Issues of effective policy design can also be explored in more depth through what would be a new conceptual lens for safety climate research. We also advocate the use of more innovative methods such as ethnography to untangle these issues and suggest that other important avenues for future research include replicating this work on lower-tier construction sites. Our findings also spotlight the need for more research in areas such as modern slavery, the grey informal workers economy and worker wellbeing.

Finally, from a practical perspective, our findings highlight the need for managers and policy makers to develop improved methods of communication around OHS policy, especially engaging with new multimedia technologies. There is also a need to improve OHS training using such technologies, behavior-based and learner-centered approaches. Safety training also need to be more regulated, robust and regular. Managers also need to increase personal accountability for OHS in construction workers through stronger management commitment and leadership, greater worker empowerment and by cultivating more open and no-blame cultures in site. Managers also need to provide a more supportive environment for workers and improve the provision of PPE to enable workers to operate safely coupled with improved monitoring and enforcement regimes. Finally, policy makers and managers need to make OHS regulations and policies less complex, more practical, more robust and more revisable, if they are to be implemented effectively on site. 

**Figure 1:**
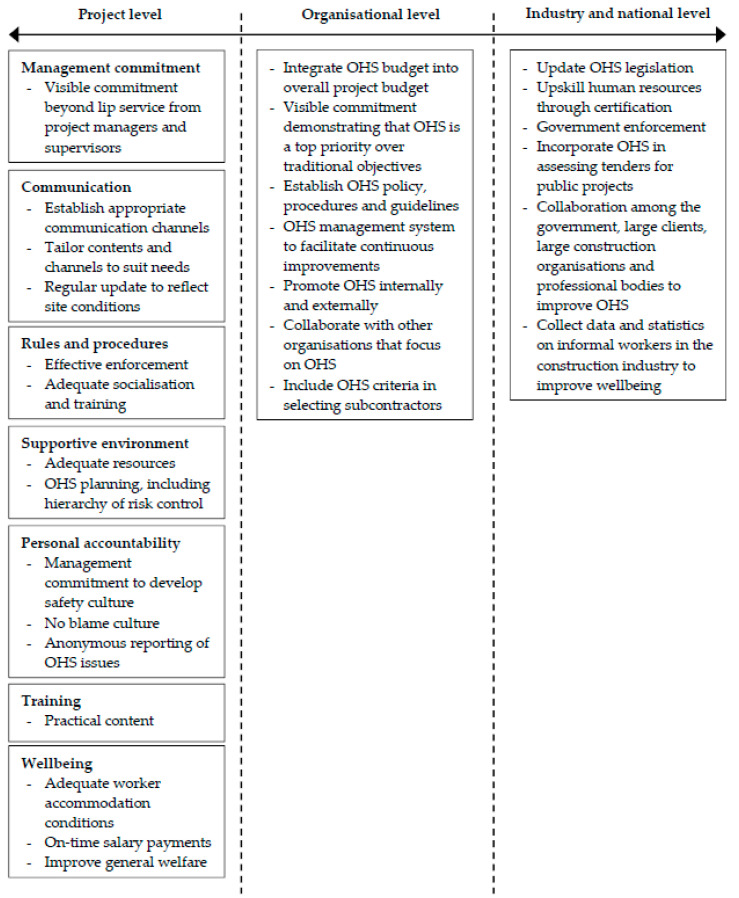
Framework for improving occupational health and safety (OHS) performance in the Indonesian construction industry.

Our results are reflected in the integrated climate framework in Figure 1 which organizes our findings and recommendations into project, organizational and industry/national levels. The framework indicates that improving OHS in the Indonesian construction industry cannot be done in isolation but only through the commitment and collaboration of numerous stakeholders across these three levels. This framework when tested and refined in other contexts, could potentially act as an important lead indicator for better safety outcomes in the Indonesian construction industry and its value should also be explored by testing in other industries and international construction industry contexts.

## Figures and Tables

**Table 1:** Sample characteristics.

Profile	Classification	Number	%
Gender	Male	302	97.11
	Female	9	2.89
Age (years)	18–24	93	29.90
	25–34	105	33.76
	35–44	74	23.79
	45–54	29	9.32
	55 and above	10	3.22
	Average	32 years	
Education	Primary school or no formal education	104	33.44
	High school	64	20.58
	Non-degree	78	25.08
	Undergraduate	60	19.29
	Postgraduate	5	1.61
Years of experience in construction	0–4	213	68.49
	5–9	57	18.33
	10–14	20	6.43
	15–19	9	2.89
	20–24	7	2.25
	25 and above	5	1.61
	Average	4.00 years	

**Table 2:** Overall safety climate score for each dimension.

Safety Climate Dimension	Mean
Management commitment	4.82
Communication	4.74
Rules and procedures	4.22
Supportive environment	4.20
Personal accountability	4.34
Training	4.64
Overall score	4.45

Note: 1 = strongly disagree, 6 = strongly agree.

**Table 3:** Management commitment dimension.

Management Commitment Items	Mean
My project manager considers the safety of employees a top priority	5.07
My project manager acts quickly to correct safety problems	5.02
My direct supervisor stops work when it is unsafe	4.93
My project manager implements corrective actions when told about unsafe behavior or conditions	4.85
My direct supervisor begins the work only when working conditions are safe	4.85
My direct supervisor pays attention to my safety	4.80
My project manager considers safety issues seriously	4.79
My project manager focuses on safety at all times, not only after accidents have occurred	4.73
My project manager expresses concern if safety procedures are not adhered to	4.36

**Table 4:** Safety communication dimension.

Safety Communication Items	Mean
Safety communication makes me pay attention on safety	5.11
Safety information is always brought to my attention by my direct supervisor	4.99
Safety communication is effective	4.98
I receive a lot of information about safety	4.96
I receive constructive suggestions if I work unsafely	4.93
Safety information is always up to date	4.91
My project manager is available for discussion when it comes to safety	4.82
My direct supervisor discusses safety issues with me	4.25
Methods used to communicate safety information are adequate	3.69

**Table 5:** Training dimension.

Training Items	Mean
The safety training provided is practical.	4.89
Potential risks and consequences are identified in safety training.	4.87
I received adequate training to perform my job safely.	4.74
The company invests a lot of time and money in safety training.	4.38
I am capable of identifying potentially hazardous situations.	4.32

**Table 6:** Personal accountability dimension.

Personal Accountability Items	Mean
A safe place to work is very meaningful for me	5.31
Safety is the number one priority for me when completing a job	5.03
A continuing emphasis on safety is important for me	5.00
I understand all the safety rules	4.89
I feel that my workplace has met the required safety standards	4.82
I am clear about my health and safety responsibilities	4.78
I am involved in implementing safety at work	4.62
I report people who ignore safety procedures	4.61
It is unlikely that I will be involved in an accident	4.42
I follow safety policy rather than simply doing what I am told to do, e.g., to work quickly and ignore safety	3.91
I can influence safety performance in my workplace	3.73
My responsibility is to work safely, including reporting co-workers who do not work safely	3.48
I am not worried about being injured on the job	3.18
It is unlikely that I will have an accident in my workplace.	3.00

**Table 7:** Rules and procedures dimension.

Rules and Procedures Items	Mean
Safety procedures are carefully followed by all	5.01
All safety rules and procedures must be followed to get the job done safely	4.61
Safety procedures are not overlooked to meet production targets	4.06
Safety rules and procedures are easy to understand	4.01
Safety requirements are not ignored to get a job done	3.97
Safety procedures are practical	3.67

**Table 8:** Supportive environment dimension.

Supportive Environment Items	Mean
My co-workers often give tips to each other on how to work safely	4.96
Employees are always encouraged to focus on safety at their workplace	4.94
No one criticizes me if I remind someone to work safely	4.70
There is punishment for behaving unsafely	4.66
I am strongly encouraged to report unsafe conditions in my workplace	4.64
There are always enough people available to get the job done safely	4.50
I receive praise for working safely	4.37
My co-workers care whether I am working safely or not	4.35
I can work safely at my workplace	4.14
Employees who report safety issues are not punished by their colleagues	3.90
I always get the tools or equipment I need to do the job safely	3.88
I am given enough time to get the job done safely	3.63
Work targets align with safety measures	3.49
Workplace conditions support my ability to work safely	3.48
My work environment reduces the possibility of accidents	3.29
